# Clinical subgroup-stratified plasma proteomic signatures improve risk prediction for myocardial infarction: SCORE2-Pro

**DOI:** 10.1186/s12933-025-03050-7

**Published:** 2026-02-02

**Authors:** Muye Tong, Minchun Zhang, Min Xu, Zhiwen Cao, Guang Ning, Weiqing Wang, Jiqiu Wang, Qian Yang, Jie Zheng

**Affiliations:** 1 Department of Endocrine and Metabolic Diseases, Shanghai Institute of Endocrine and Metabolic Diseases, Ruijin Hospital, Shanghai Jiao Tong University School of Medicine, Shanghai, China; 2 Shanghai National Clinical Research Center for Endocrine and Metabolic Diseases, Key Laboratory for Endocrine and Metabolic Diseases of the National Health Commission of the PR China, Shanghai National Center for Translational Medicine, Shanghai Digital Medicine Innovation Center, Lifecycle Health Management Center, Ruijin Hospital, Shanghai Jiao Tong University School of Medicine, Shanghai, China; 3 MRC Integrative Epidemiology Unit (IEU), Bristol Medical School, University of Bristol, Augustine’s Courtyard, Orchard Lane, Bristol, BS1 5DS UK

**Keywords:** Myocardial infarction, Plasma proteomics, SCORE2, Subgroup analysis, Risk prediction

## Abstract

**Background:**

Myocardial infarction (MI) remains a leading cause of global mortality, with risk varying substantially across demographic and clinical subgroups. Although SCORE2 is widely implemented for cardiovascular risk stratification, the extent to which clinical subgroup specific plasma proteomics can further refine personalized MI risk prediction remains uncertain.

**Methods:**

SCORE2-Pro, a clinical subgroup-stratified plasma proteome prediction model was built stratified by sex, age, smoking status, non-high-density lipoprotein (non-HDL) cholesterol, and systolic blood pressure. In 51,010 UK Biobank participants (aged 40–69 years; 54.9% female) without MI at baseline, 70% were used for model development, and the remaining 30% for an internal hold-out validation. We used light gradient boosting machine classifiers and Cox proportional hazards models to identify top-predictive protein combinations and stratification strategies for MI.

**Results:**

The SCORE2-Pro model revealed distinct and highly effective protein panels for each subgroup. Compared with the clinical model, SCORE2-Pro remarkably enhanced predictive performance across demographic and clinical subgroups. A 9-protein model in females improved AUC by + 0.061 (*P* = 1.51 × 10^−3^), with a net reclassification index (NRI) of + 0.125 (*P* = 2.69 × 10^−6^). Similarly, a 7-protein model for middle-aged subpopulation demonstrated an AUC improvement of + 0.036 (*P* = 0.033) with an NRI of + 0.127 (*P* = 8.09 × 10^−11^). Notably, in high-risk populations, SCORE2-Pro model significantly improved reclassification performance compared with the clinical model (NRI: + 0.121, *P* = 0.020).

**Conclusions:**

By adopting a clinical subgroup stratification approach using factors derived from SCORE2, we identified subgroup-specific proteomic signatures for MI that considerably improve predictive accuracy and reclassification beyond traditional clinical models.

**Supplementary Information:**

The online version contains supplementary material available at 10.1186/s12933-025-03050-7.

## Introduction

Cardiovascular diseases (CVDs) remain the leading cause of morbidity and mortality worldwide, with myocardial infarction (MI) being a major contributor to its clinical burden [1–3]. Early identification of individuals at elevated risk is central to effective primary prevention and is a priority in current cardiovascular guidelines [4]. The SCORE2 algorithm, recommended by the European Society of Cardiology, is widely used in clinical practice [5]. It predicts the 10-year risk of CVDs based on conventional risk factors, including age, sex, smoking status, systolic blood pressure, and lipid levels [6]. However, substantial heterogeneity in MI risk persists even among individuals with similar SCORE2 profiles, underscoring the need for more biologically informative markers that improve precision in primary prevention [7].

Circulating proteins integrate signals from inflammation, metabolic dysregulation, and vascular injury, making them promising biomarkers to enhance cardiovascular risk prediction [8]. Studies have demonstrated that integrating proteomic data with clinical factors can improve the discrimination of individuals at risk for CVDs beyond traditional risk factors [9–12]. For example, a data-driven protein model comprising 114 markers, when combined with SCORE2, has been shown to significantly improve the prediction of major adverse cardiovascular events (MACE) [10]. However, most studies have applied generalized models across the entire study population, overlooking the biological heterogeneity across clinical subgroups. Move one step forward, a recent study improved risk prediction accuracy by incorporating sex-specific proteomic profiles into the SCORE2 model, with greater enhancements observed in men compared to women [13]. A recent large-scale cohort study further underscored the presence of sex-specific associations between circulating proteins and incident MI, reinforcing the importance of subgroup-specific proteomic models in risk prediction [14]. These studies imply that subgroup-specific proteomic models may offer superior predictive performance over the general proteomics models. In summary, developing a novel MI risk prediction model by integrating the risk stratification strategy promoted by SCORE2 with large-scale proteomic data may offer a timely opportunity to inform personalized MI risk prevention.

This study aims to identify proteomic markers for MI and assess their potential to enhance risk prediction in clinical factors stratified subpopulations. We conducted a comprehensive proteomic analysis in 51,010 participants from the UK Biobank, to systematically develop subgroup-specific predictive models aligned with the five key stratification variables used in SCORE2, including sex, age, smoking status, systolic blood pressure (SBP), and non-high-density lipoprotein (HDL) cholesterol.

## Methods

### Study population

This study utilized individual-level proteomic and phenotypic data from the UKB. The UKB is a large, prospective cohort study comprising over 500,000 participants aged 40–69 years, recruited between 2007 and 2010 across 22 assessment centers in England, Wales, and Scotland [15, 16] The UKB was conducted in accordance with the Declaration of Helsinki, with all participants providing written informed consent. In this study, participants without plasma proteomic data and with a history of MI at baseline were excluded, and 51,010 participants were included in subsequent analyses (Fig. S1).

### Definitions of outcomes

The disease outcomes were incident MI and its subtypes non–ST-segment elevation MI (NSTEMI) and ST-segment elevation MI (STEMI). The date of MI diagnosis and subtypes of MI were obtained from the algorithmically defined MI events conducted by the UK Biobank Cardiac Outcomes Group (fields 42,000–42,005) [17]. In detail, MI events and their subtypes were identified through a combination of data sources, including self-reported medical conditions at baseline (field 20,002), hospital admissions (Data-Coding 2000), and ICD-10 codes in death register records (fields 40,001–40,002). These sources were integrated using predefined algorithms designed to maximize positive predictive value. The date of diagnosis was defined as the earliest recorded date across available sources.

### Covariates

Demographic and clinical variables were obtained or derived from UKB baseline assessment data. Age (field 21,003), sex (field 31), ethnic background (field 21,000), smoking status (field 20,116), qualifications (field 6138), and diabetes diagnosed by doctor (field 2443) were collected via standardized questionnaires. Diagnoses were coded using hospital records (ICD-10 codes; field 41,270). Body mass index (BMI, field 21,001) was calculated from measured height and weight. Systolic and diastolic blood pressure (fields 4080 and 4079) were measured twice using an automated device on the left upper arm during each assessment visit. The average across visits was used in the analysis. HDL cholesterol (field 30,760), low-density lipoprotein (LDL) cholesterol (field 30,780), cholesterol (field 30,690), triglycerides (field 30,870), creatinine (field 30,700), and C-reactive protein (field 30,710) were quantified from serum samples using the Beckman Coulter AU5800 platform. Missing values of the clinical variables were imputed with missRanger [18] (R package version 2.6.1, an efficient implementation of the missForest algorithm [19]).

### Plasma proteomic data

The UK Biobank Pharma Proteomics Project (UKB-PPP) conducted a thorough analysis of blood-based proteomic data. Blood samples were collected at the baseline visit from 2007 to 2010, and a subsample of approximately 55,000 participants was primarily randomly selected from participants at 22 local assessment centers across the UK. These samples were collected using EDTA vacutainers, and the plasma was then divided into aliquots and stored at − 80 °C until further processing. The proteomic profiling was performed on the Olink® Explore 3072 platform in Sweden, utilizing the Proximity Extension Assay in combination with Next-Generation Sequencing [20]. The protein levels were quantified and expressed as Normalized Protein eXpression (NPX) values. All sample characteristics and clinical data were blinded to the investigators. Rigorous quality control procedures were implemented with detailed protocols [21]. The sample selection, processing, and quality control protocols for the Olink assay were also described in previous publications [22–24]. Technical variation, including batch and plate effects, has been systematically examined in the UKB-PPP dataset previously [23]. In total, 2923 unique proteins were captured. After excluding proteins with missing data of over 25%, a total of 2919 remained for the subsequent analysis.

### Statistical analysis

#### Observational association analysis

##### Cox proportional hazards regression models

Cox proportional hazards regression models were employed to adjust for baseline covariates for each plasma protein NPX value (scaled) in relation to the outcomes of incident MI and NSTEMI. Given the limited number of STEMI cases (n < 500), this disease subtype was excluded from subsequent analyses. Age, sex, and education years were adjusted in Model 1, while Model 2 further adjusted for diabetes diagnosis, smoking status (never, previous, and current), BMI, SBP, diastolic blood pressure, total cholesterol, triglycerides, HDL cholesterol, LDL cholesterol, C-reactive protein, and estimated glomerular filtration rate. Statistical significance was determined using Bonferroni corrections (*P* < 0.05), accounting for the total number of proteins tested. The results of these analyses were presented as hazard ratio (HR), 95% confidence intervals (CIs), and corresponding *P* values (Supplementary Table S1).

##### Kaplan–Meier survival analysis

Kaplan–Meier survival analysis was conducted to illustrate the clinical progression of MI events over time. Participants were categorized into high and low baseline groups based on levels of the selected protein, using the optimal cutoff determined via the Youden index. The number of individuals whose observation period extended 15 years was small and thus treated as 15 years. Cox proportional hazards models, adjusted for age, sex, education, diabetes diagnosis, smoking status (never, previous and current), BMI, SBP, diastolic blood pressure, total cholesterol, triglycerides, HDL cholesterol, LDL cholesterol, C-reactive protein, and estimated glomerular filtration rate, were then utilized to estimate the prognostic differences linked with dichotomized protein concentrations. The analysis was conducted using the R package survminer.

#### Risk prediction analysis

For all risk prediction analysis, we randomly divided the 51,010 UKB participants into a derivation set (70%) used for model training and a validation set (30%) used for performance assessment. The two sets were comparable in demographic characteristics, clinical risk factors, and MI incidence in both the general population and clinical subgroups (Supplementary Table S2).

##### Protein importance ranking in MI and NSTEMI prediction models

To identify crucial proteins, we performed a methodical two-step process consisting of variable importance ranking and sequential forward selection, as detailed in a previous study [25]. First, significant proteins passed the Bonferroni corrected threshold from the Cox proportional hazards regressions in Model 2 were fed into a preliminary trained light gradient boosting machine (LGBM) classifier [26]. The contribution of each protein to the model’s predictive performance was utilized to rank importance in predicting the onset of MI. Subsequently, we employed a sequential forward selection approach, systematically integrating proteins into the newly developed empty LGBM classifier one by one based on their importance ranking. The cumulative area under the curve (AUC) for predicting events was calculated. Optimal performance was defined as the point where four consecutive DeLong tests showed no further improvement and the AUC increase was < 0.003, indicating no meaningful gain from adding more proteins. The selected proteins with highest priority score were then visualized using SHapley Additive exPlanations (SHAP) plots (shapviz, R package version 0.9.3). Protein importance ranking was separately conducted for MI and NSTEMI cases within the derivation set.

##### Assessing predictive accuracy in predicting MI and NSTEMI

Receiver operating characteristic (ROC) analyses were conducted to assess the predictive accuracy of the selected important proteins or protein panels identified using the LGBM model for predicting MI and NSTEMI in the untouched 30% validation set. To compare the AUC values between different models, DeLong tests were performed using the R package pROC.

The model establishment and evaluation were conducted through internal leave-one-out cross-validation. We split the dataset into ten folds based on geographical locations (East Midlands, London, North East, North West, Scotland, South East, South West, Wales, West Midlands, and Yorkshire and Humber) of the 22 assessment centers. Each iteration used nine folds for training and one for testing, repeated ten times with rotated folds. To assess model stability and potential overfitting, we further performed bootstrap optimism correction with 500 resamples. The optimism-corrected AUCs are provided in Supplementary Table S3.

##### Clinical subgroup-specific protein associations

To explore heterogeneity in the prognostic value of key proteins across clinical subgroups, we divided the overall UKB-PPP participants into two subgroups based on each of the five SCORE2 clinical stratification variables: sex (female vs. male), age (< 60 years [middle-aged] vs. ≥ 60 years [elderly]), smoking status (never smokers [non-smoking] vs. previous/current smokers [smoking]), SBP (< 140 mmHg vs. ≥ 140 mmHg), and non-HDL cholesterol (< 4 mmol/L vs. ≥ 4 mmol/L), and estimated the HRs and 95%CIs for the top ranked proteins identified by the general prediction model in each subgroup. The SBP threshold reflects the 140 mmHg boundary defining hypertension in European hypertension guidelines [27]. The non-HDL-C threshold was chosen based on a large population-based study, making 4 mmol/L a practical division between lower- and higher-risk for cardiovascular disease [28]. Cox proportional hazards models (Model 2) were applied separately within each subgroup. To compare the strength of association between subgroups, pairwise Z-tests were used to evaluate differences in HRs.

##### SCORE2-Pro, clinical subgroup-specific model construction

We developed a subgroup-specific proteomic model, SCORE2-Pro, which aligned with five SCORE2 clinical stratification variables: sex, age, smoking status, SBP, and non-HDL cholesterol. We used protein profiles to predict MI risk in each subgroup. For each subgroup, the 70% derivation set was used to perform the protein importance ranking. All significant proteins passed the multiple correction threshold in the Cox model in each subgroup were used as the input for a preliminary LGBM model to rank their importance in predicting MI risk. A sequential forward selection procedure was then applied: proteins were added iteratively to an empty LGBM classifier based on their ranked importance. At each step, the cumulative AUC was computed. Optimal protein panels were defined as the point where four consecutive DeLong tests indicated no further performance gain and the incremental AUC increase was < 0.003. These subgroup-specific protein combinations were subsequently validated in the 30% internal hold-out validation data set, and model performance was compared against that of the clinical SCORE2 model using DeLong tests. In addition, the net reclassification improvement (NRI) was calculated to assess the added value of proteomic models over the clinical SCORE2 model.

##### Reclassification performance in clinical subgroups and high-risk populations

We evaluated model performance in a high-risk subgroup defined as male participants who were current or previous smokers with SBP ≥ 140 mmHg and non-HDL cholesterol ≥ 4 mmol/L. This definition was informed by the SCORE2 risk stratification framework, which identifies male, elderly, smoking, elevated systolic blood pressure, and high non–HDL cholesterol as key contributors to elevated cardiovascular risk [6]. Consistent with this classification, the observed incidence of MI in this composite subgroup was ~ 9% (Supplementary Table S4), markedly higher than the ~ 4% (2014/51,010) incidence observed in the overall study population. The proteomic model used for this analysis was constructed by combining proteins identified from the corresponding subgroup-specific models. Model performance was assessed by NRI. Categorical NRI was calculated using a 9% 10-year risk threshold, based on the observed incidence of MI in this subgroup. NRI was reported separately for MI events and non-events. NRI calibration metrics are presented in Fig. S2 and Supplementary Table S5–S6.

Data analysis and visualizations were performed using Python (v.3.9) and R (v.4.0.3). Statistical significance was set at two-tailed *P* < 0.05. We also provided a complete ML-CLAIM checklist for this study to enhance methodological transparency (Supplementary Table S7) [29].

## Results

### Baseline characteristics of participants

The baseline characteristics are summarized in Table 1. In brief, a total of 51,010 individuals without prior MI at baseline were included (median age: 58 years [IQR 50–63]; 54.9% female). During a median follow-up of 13.8 years, 2014 individuals (3.9%) experienced incident MI, including 1103 cases of NSTEMI and 472 cases of STEMI (Fig. S1). Compared with participants who remained MI-free, those individuals with MI were older (median age: 62 vs. 58 years), more likely to be male (65.1% vs. 44.3%), and exhibited a higher cardiovascular risk profile as defined by SCORE2 algorithm. In addition, MI cases had a higher prevalence of other metabolic comorbidities, including diabetes and obesity, as well as increased C-reactive protein and reduced eGFR at baseline (all *P* < 0.001) (Table 1). These associations were consistent across both NSTEMI and STEMI subtypes (Table 1).

**Table 1:** Baseline characteristics of UK Biobank participants included in the study. Continuous data and categorical variables are shown as median [interquartile range] and number (percentage), respectively. *P* values were calculated using t-tests for continuous variables and χ^2^ tests for categorical variables between each incident group (MI, NSTEMI, or STEMI) and the control group

Participants’ characteristics	Overall	Control	Incident MI		Incident NSTEMI		Incident STEMI	
	N = 51,010	N = 48,996	*N* = 2,014	*P* value	*N* = 1,103	*P* value	*N* = 472	*P* value
Age, years	58 [50, 63]	58 [50, 63]	62 [57, 66]	9.48 × 10^−123^	62 [57, 66]	3.49 × 10^−68^	61 [54, 65]	1.49 × 10^−12^
Sex (female)	27,992 (54.9)	27,289 (55.7)	703 (34.9)	1.95 × 10^−75^	421 (38.2)	2.68 × 10^−29^	137 (29.0)	6.05 × 10^−30^
Education, years	15 [10, 20]	15 [10, 20]	15 [7, 19]	2.97 × 10^−41^	13 [7, 19]	3.25 × 10^−28^	15 [7, 19]	2.80 × 10^−06^
Diabetes (%)	2694 (5.3)	2422 (4.9)	273 (13.5)	1.28 × 10^−46^	149 (13.5)	8.27 × 10^−26^	41 (8.7)	1.82 × 10^−03^
Current smoker (%)	5349 (10.5)	4991 (10.2)	358 (17.8)	7.11 × 10^−24^	196 (17.8)	1.69 × 10^−13^	101 (21.4)	4.03 × 10^−12^
Body mass index	26.7 [24.2, 29.9]	26.7 [24.1, 29.8]	28.0 [25.3, 31.2]	5.47 × 10^−36^	27.9 [25.3, 31.2]	2.70 × 10^−17^	27.6 [25.1, 30.2]	6.18 × 10^−05^
Systolic blood pressure, mmHg	138 [126, 152]	138 [125, 152]	145 [132, 158]	1.11 × 10^−50^	146 [133, 160]	1.13 × 10^−33^	145 [132, 157]	2.41 × 10^−13^
Diastolic blood pressure, mmHg	82 [75, 89]	82 [75, 89]	83 [76, 91]	9.26 × 10^−10^	84 [76, 91]	1.39 × 10^−06^	84 [78, 91]	1.93 × 10^−06^
Total cholesterol, mmol/L	5.6 [4.9, 6.4]	5.6 [4.9, 6.4]	5.5 [4.6, 6.4]	1.16 × 10^−10^	5.6 [4.7, 6.4]	7.56 × 10^−04^	5.9 [5.0, 6.7]	9.70 × 10^−06^
Triglyceride, mmol/L	1.5 [1.0, 2.1]	1.5 [1.0, 2.1]	1.7 [1.3, 2.5]	1.86 × 10^−42^	1.8 [1.2, 2.5]	2.00 × 10^−24^	1.8 [1.3, 2.5]	1.44 × 10^−14^
HDL cholesterol, mmol/L	1.4 [1.2, 1.7]	1.4 [1.2, 1.7]	1.3 [1.1, 1.5]	7.11 × 10^−79^	1.3 [1.1, 1.5]	6.73 × 10^−40^	1.2 [1.1, 1.5]	3.30 × 10^−22^
LDL cholesterol, mmol/L	3.5 [2.9, 4.1]	3.5 [2.9, 4.1]	3.5 [2.8, 4.1]	1.29 × 10^−03^	3.5 [2.9, 4.1]	3.99 × 10^−01^	3.9 [3.2, 4.5]	2.62 × 10^−11^
C-reactive protein, mg/L	1.3 [0.7, 2.8]	1.3 [0.7, 2.8]	1.9 [0.9, 4.0]	4.21 × 10^−41^	1.9 [0.9, 4.1]	2.23 × 10^−26^	1.8 [0.9, 3.5]	7.73 × 10^−08^
eGFR, mL/min/1.73m^2^	100.7 [85.3, 118.5]	101.1 [85.8, 118.8]	90.2 [76.6, 107.2]	3.70 × 10^−76^	90.8 [77.4, 109.3]	9.83 × 10^−32^	89.4 [76.7, 106.0]	2.27 × 10^−21^

MI, myocardial infarction; NSTEMI, non-ST-elevation myocardial infarction; STEMI, ST-elevation myocardial infarction; HDL, high-density lipoprotein; LDL, low-density lipoprotein; CRP, C-reactive protein; eGFR, estimated glomerular filtration rate

### Plasma proteomic biomarkers associated with MI

**Fig. 1:**
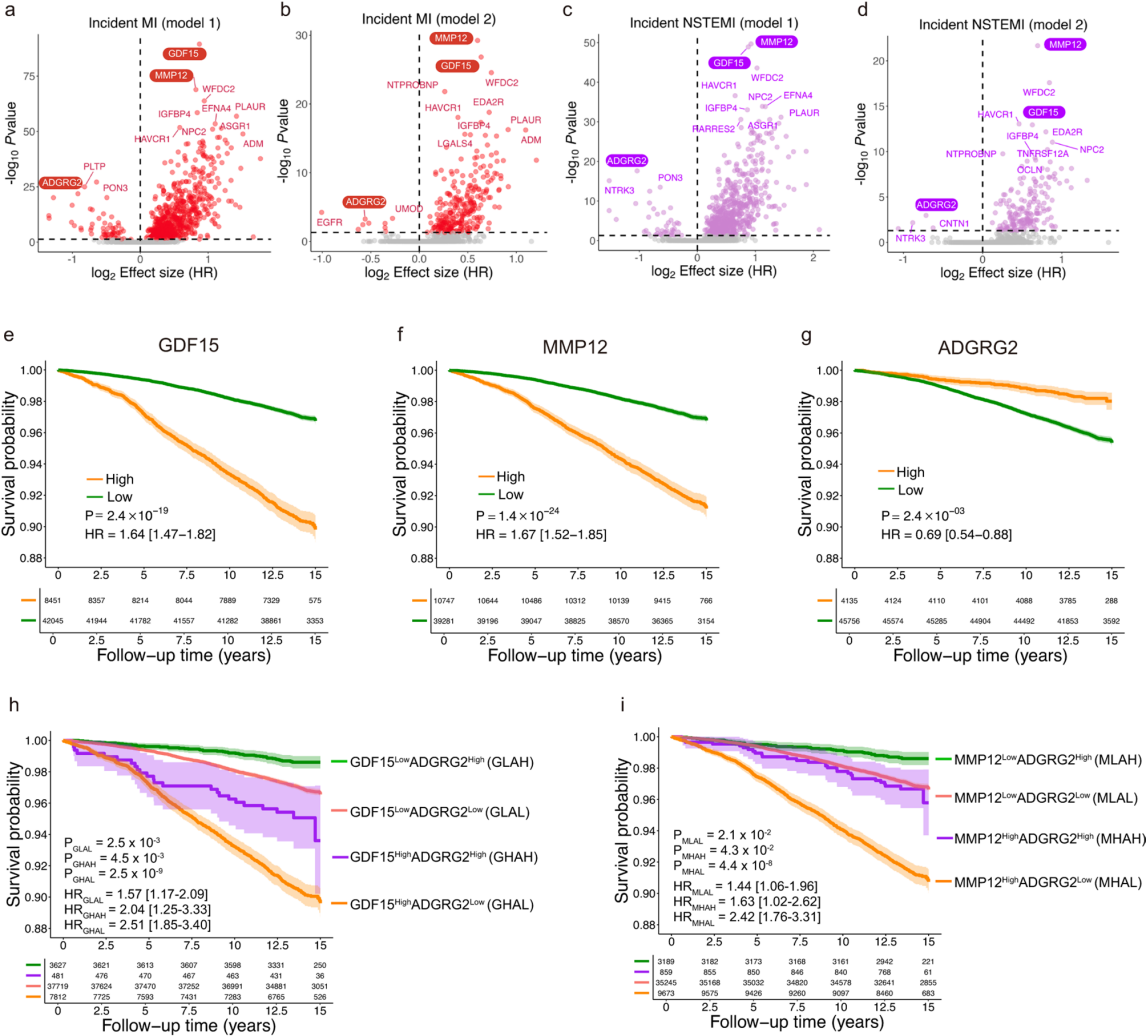
Baseline protein signatures associated with myocardial infarction.** a**–**d** Volcano plots show log2 Hazard ratios (HR) on the x-axis and -log10 (*P* value) on the y-axis for protein signatures associated with myocardial infarction (MI) and non–ST-segment elevation MI (NSTEMI). The black dashed line indicates significance after Bonferroni correction. Top 10 proteins with log2 HR > 0 and top 3 with log2 HR < 0 are labeled. Model 1: adjusted for age, sex, and education; Model 2: further adjusted for diabetes diagnosis, smoking status, body mass index, systolic blood pressure (SBP), diastolic blood pressure, total cholesterol, triglycerides, high-density lipoprotein (HDL) cholesterol, low-density lipoprotein (LDL) cholesterol, C-reactive protein, and estimated glomerular filtration rate.** e**–**i** Unadjusted Kaplan–Meier curves show MI incidence over time. The numbers presented below each curve represent the number of participants remaining at risk at the corresponding follow-up time points. Cox models adjusted for the covariates in Model 2 were used to estimate the association between baseline protein levels and MI risk. Shaded regions represent standard errors. HR and P values were calculated for each group compared to the GDF15^Low^ADGRG2^High^ group (**h**) or MMP12^Low^ADGRG2^High^ group (**i**)

After adjusting for demographic covariates (Model 1), 838 plasma proteins were significantly associated with MI (Fig. 1a, Supplementary Table S1). After further adjustment for cardiovascular risk factors (Model 2), 281 proteins remained significant (Fig. 1b, Supplementary Table S1). For NSTEMI, 676 and 185 proteins were significantly associated with the event risk in Model 1 and Model 2, respectively (Fig. 1c, d, Supplementary Table S1).

Among 2919 plasma proteins assessed, growth differentiation factor 15 (GDF15), matrix metalloproteinase 12 (MMP12), and adhesion G protein–coupled receptor G2 (ADGRG2) showed consistent and robust associations with both MI and NSTEMI across all models (Fig. 1a–d), and were thus selected for subsequent survival analysis. Elevated baseline levels of GDF15 and MMP12 were strongly associated with increased risk of MI (GDF15: HR = 1.64 [95% CI: 1.47–1.82], *P* = 2.4 × 10^–19^; MMP12: HR = 1.67 [1.52–1.85], *P* = 1.4 × 10^–24^) (Fig. 1e, f). In contrast, ADGRG2 was associated with reduced risk (HR = 0.69 [0.54–0.88], *P* = 2.4 × 10^–3^) (Fig. 1g), representing a novel protective marker not previously linked to MI. Similar patterns were also observed for NSTEMI (Fig. S3). Notably, individuals with simultaneously elevated GDF15 or MMP12 and reduced ADGRG2 levels exhibited substantially higher MI risk (GDF15^high^ADGRG2^low^: HR = 2.51 [95% CI: 1.85–3.40]; MMP12^high^ADGRG2^low^: HR = 2.42 [95% CI: 1.76–3.31]) (Fig. 1h, i), exceeding the risk associated with individual protein.

### Identification of a seven-protein panel for MI prediction in the general population

**Fig. 2:**
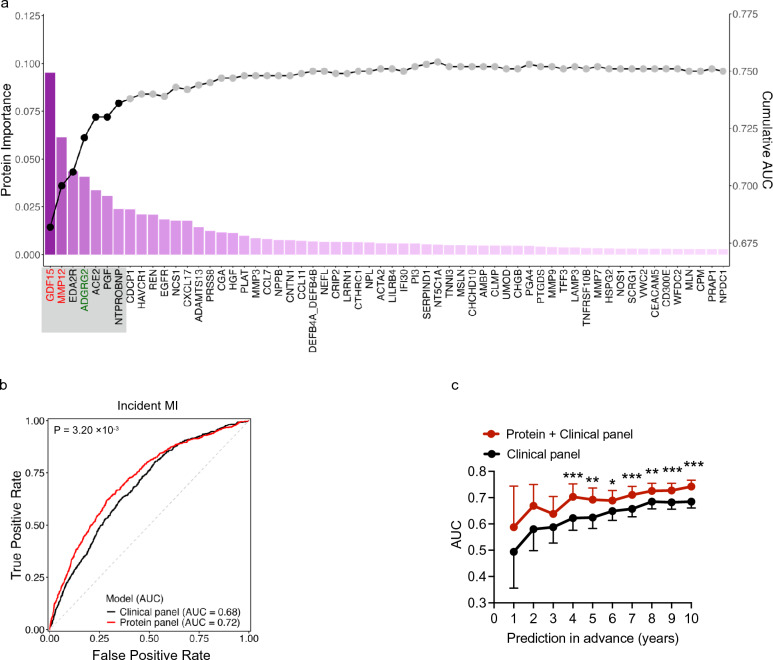
Performance of the seven-protein panel for MI prediction. **a** Sequential forward selection for candidate proteins in the derivation set. The bar plot illustrates the importance of sorted proteins based on their contributions to predicting future MI (left axis). The line plot shows cumulative Area Under the Curve (AUC) values (right axis). Seven proteins selected for the subsequent MI prediction model are highlighted with gray rectangular backgrounds. Risk proteins (GDF15 and MMP12) were labeled in red, and the protective protein (ADGRG2) was labeled in green. **b** Receiver operating characteristic (ROC) curves and AUC values show the performance of different variable models for predicting incident MI in the validation set. The protein panel includes top-ranked seven proteins identified in **a**. **c** Line plots displaying the dynamic AUC for predicting incident MI over time in the validation set. DeLong tests were used to compare the AUC values between models. Statistical significance is denoted as follows: * *P* < 0.05, ** *P* < 0.01, *** *P* < 0.001

Through our machine learning model, we identified a panel of 7 proteins (GDF15, MMP12, EDA2R [ectodysplasin A2 receptor], ADGRG2, ACE2 [angiotensin-converting enzyme 2], PGF [placental growth factor], and NT-proBNP [N-terminal pro-B-type natriuretic peptide]) that significantly increased predictive performance within the derivation set (Fig. 2a, Fig. S4a, Supplementary Table S8). Inclusion of additional proteins did not enhance model accuracy.

In the validation set, this protein panel demonstrated strong predictive performance with an AUC of 0.72, surpassing that of the clinical risk model (AUC = 0.68, *P* = 3.20 × 10^−3^; Fig. 2b, Supplementary Table S9). When integrated with the clinical model, the combined model significantly enhanced MI prediction starting at the fourth year of follow-up (Fig. 2c). At 10 years prior to the event, the AUC reached 0.74 for the combined panel, compared to 0.68 for the clinical model (*P* = 3.58 × 10^−7^; Fig. 2c, Supplementary Table S10). Applying the same procedure to NSTEMI yielded a distinct set of predictive proteins for this MI subtype, which the proteomic model also improved 10-year predictive performance (Fig. S5, Supplementary Table S9).

### SCORE2-Pro, a subgroup-specific prediction model

**Fig. 3:**
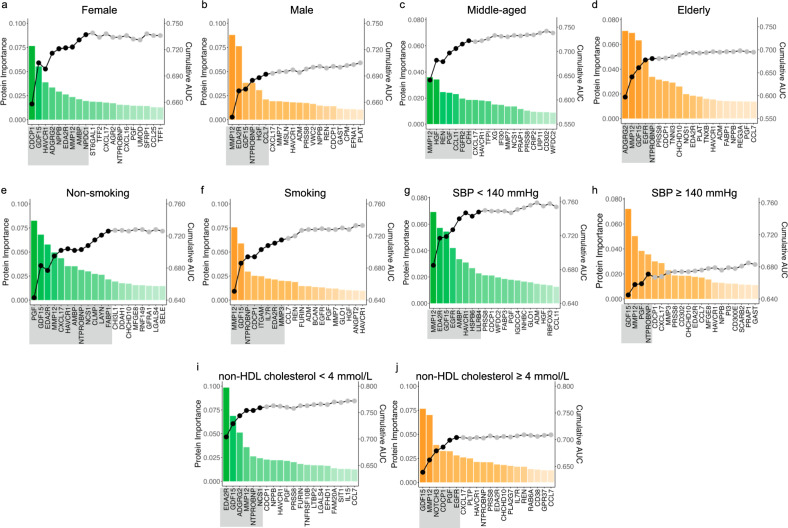
Sequential forward selection of protein panels for MI prediction in various subgroups. Sequential forward selection for candidate proteins in subgroups stratified by sex **a**, **b**, age **c**, **d**, smoking status **e**, **f**, SBP **g**, **h**, and non-HDL cholesterol **i**, **j** in the derivation set. The bar plot illustrates the importance of sorted proteins based on their contributions to predicting future MI (left axis). The line plot shows cumulative AUC values (right axis). Proteins selected for MI prediction model in each subgroup are highlighted with gray rectangular backgrounds

Moreover, analysis of the 7 proteins revealed heterogeneous associations with MI across the 5 demographic and clinical subgroups defined by SCORE2 risk stratification, suggesting the need for a protein-based model stratified by clinical subgroups (Fig. S6). Therefore, we developed a subgroup-specific proteomic model, SCORE2-Pro, which applied a similar stratification process used by SCORE2, including sex, age, smoking status, SBP, and non-HDL cholesterol as startification variables (Fig. 3, Fig. S4b–k, S7, Supplementary Table S11). These specific models achieved superior performance compared to the conventional clinical model across all subgroups (Table 2). For instance, in females, a 9-protein panel improved AUC from 0.638 to 0.699 (*P* = 1.51 × 10^−3^), with a calibrated NRI of + 0.125 [95% CI: 0.085–0.164] (*P* = 2.69 × 10^−6^). In males, a 6-protein model increased AUC by + 0.073 (from 0.603 to 0.676, *P* = 1.27 × 10^−7^), yielding a calibrated NRI of + 0.061 [0.034–0.087] (*P* = 1.93 × 10^−10^). Similar performance gains were observed in other subgroups, including middle-aged individuals (+ 0.036 AUC, + 0.127 NRI) and participants with elevated SBP (+ 0.066 AUC, + 0.069 NRI). Notably, individuals with non-HDL cholesterol < 4 mmol/L—traditionally considered as low-risk—also benefited from proteomic reclassification (+ 0.094 AUC, + 0.087 NRI) (Table 2). We also compared the subgroup-specific protein models with the general protein model within each clinical subgroup. The specific model achieved markedly better performance in females, with an AUC increase of 0.031 (from 0.668 to 0.699; *P* = 3.29 × 10^−3^), while several other specific protein models demonstrated similar improvement with fewer proteins included in the models (Supplementary Table S12).

**Table 2:** Area under curve and net reclassification index of 2 models in the validation set. Model performance was evaluated in demographic and clinical subgroups using the validation cohort. The net reclassification indices were calculated based on a 5% risk threshold relative to the clinical model

Model	Subpopulation	No. of protein markers	Risk discrimination				Reclassification performance			
			AUC (∆AUC)	Lower 95% CI	Upper 95% CI	*P* value	NRI	Lower 95% CI	Upper 95% CI	*P* value
Clinical model										
Sex	Female	–	0.638	0.598	0.677					
	Male	–	0.603	0.576	0.630					
Age	Middle-aged	–	0.672	0.634	0.709					
	Elderly	–	0.606	0.577	0.635					
Smoke	Non-smoking	–	0.662	0.632	0.691					
	Smoking	–	0.650	0.621	0.679					
Systolic blood pressure	< 140 mmHg	–	0.667	0.634	0.701					
	≥ 140 mmHg	–	0.625	0.597	0.654					
Non-HDL cholesterol	< 4 mmol/L	–	0.660	0.629	0.691					
	≥ 4 mmol/L	–	0.671	0.643	0.700					
Specific protein model										
Sex	Female	9	0.699 (+ 0.061)	0.662	0.736	1.51 × 10^−3^	+ 0.125	0.085	0.164	2.69 × 10^−06^
	Male	6	0.676 (+ 0.073)	0.651	0.702	1.27 × 10^−7^	+ 0.061	0.034	0.087	1.93 × 10^−10^
Age	Middle-aged	7	0.708 (+ 0.036)	0.671	0.745	3.30 × 10^−2^	+ 0.127	0.088	0.169	8.09 × 10^−11^
	Elderly	5	0.668 (+ 0.062)	0.640	0.696	2.60 × 10^−5^	+ 0.088	0.060	0.116	2.05 × 10^−11^
Smoke	Non-smoking	12	0.691 (+ 0.029)	0.660	0.722	4.04 × 10^−2^	+ 0.118	0.080	0.154	1.96 × 10^−10^
	Smoking	8	0.704 (+ 0.054)	0.677	0.731	8.52 × 10^−5^	+ 0.057	0.029	0.087	2.87 × 10^−04^
Systolic blood pressure	< 140 mmHg	8	0.732 (+ 0.065)	0.701	0.763	3.84 × 10^−5^	+ 0.132	0.097	0.168	6.44 × 10^−13^
	≥ 140 mmHg	4	0.691 (+ 0.066)	0.664	0.719	4.13 × 10^−7^	+ 0.069	0.042	0.097	1.03 × 10^−06^
Non-HDL cholesterol	< 4 mmol/L	6	0.754 (+ 0.094)	0.727	0.781	3.02 × 10^−10^	+ 0.087	0.054	0.118	3.14 × 10^−08^
	≥ 4 mmol/L	6	0.698 (+ 0.027)	0.669	0.727	3.66 × 10^−2^	+ 0.068	0.035	0.097	3.66 × 10^−06^

AUC, area under the curve; NRI, net reclassification index; CI, confidence interval; HDL, high-density lipoprotein

### Subgroup-specific proteomic risk prediction patterns for MI

**Fig. 4:**
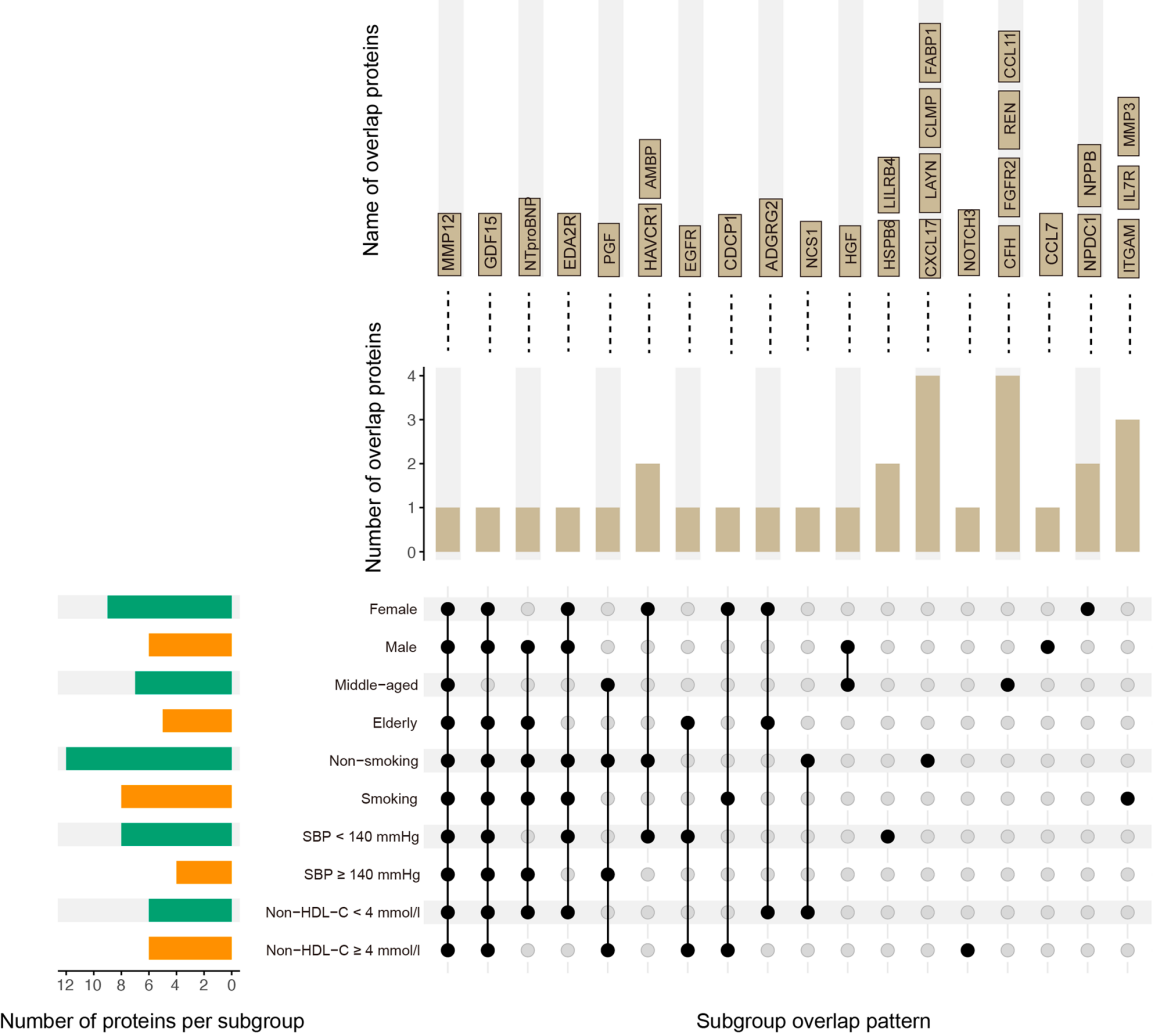
Subgroup-specific plasma proteomic signatures for MI prediction. UpSet plot illustrating the distribution of proteins identified across ten clinical subgroups defined by SCORE2 stratification variables (sex, age, smoking status, SBP, and non-HDL cholesterol). Bars at the top represent the number of proteins associated with each subgroup intersection, with corresponding protein names shown above. Connected black dots indicate shared proteins across multiple subgroups, whereas the single dots denote subgroup-unique proteins. Horizontal bars on the left display the total number of proteins identified in each subgroup

To systematically evaluate the subgroup-specific predictive models, we summarized the selected proteins across ten clinical subgroups defined by SCORE2 (SCORE2-Pro) and visualized their intersections using an UpSet plot (Fig. 4). In total, 29 proteins were selected, with the number per subgroup ranging from 4 (specific panel for SBP ≥ 140 mmHg) to 12 (specific panel for non-smoking) (Fig. 4, Supplementary Table S13). Four proteins (i.e., GDF15, MMP12, EDA2R, and NT-proBNP) were shared by at least six subgroups and also formed the core of the 7-protein panel identified in the general population (Fig. 2a, 4). Among them, GDF15 was selected in 9 out of 10 subgroups, consistent with its established role as a robust biomarker of cardiovascular risk [10, 12, 30]. Notably, 17 proteins showed subgroup-specific associations. For example, integrin alpha-M 1 (ITGAM), interleukin-7 receptor (IL7R), and stromelysin-1 (MMP3) were prominently featured in smokers (Fig. 4), while neurogenic locus notch homolog protein 3 (NOTCH3) was more specific in those with elevated non-HDL cholesterol (Fig. 4), underscoring the importance of subgroup-specific proteomic profiling in MI risk prediction.

### Reclassification improvements in high-risk populations

**Fig. 5:**
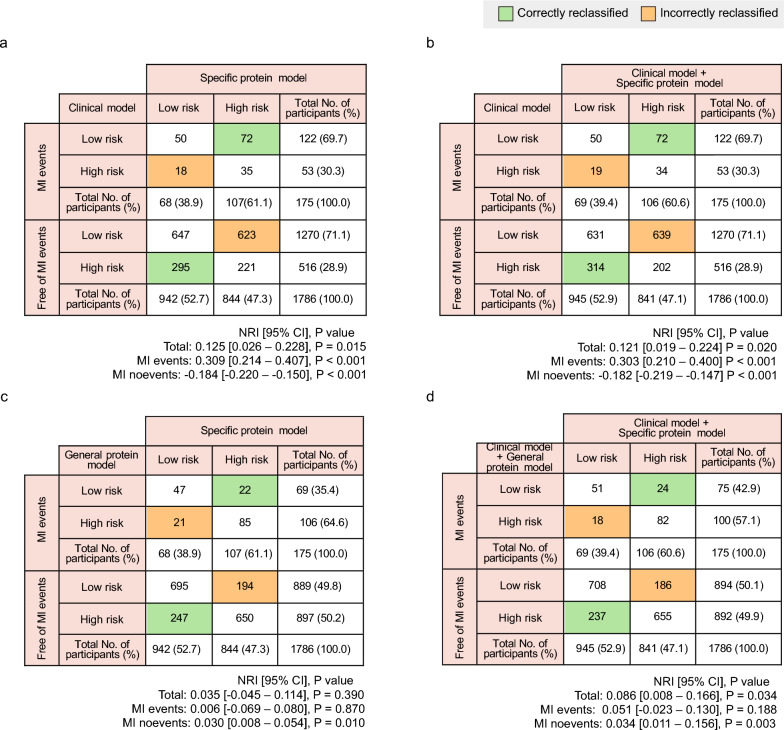
Reclassification in the high-risk population. The figure shows reclassification results in the high-risk population (male smokers with elevated SBP and non-HDL cholesterol). The reclassification tables compare the performance of SCORE2-Pro (**a**, **b**) and general protein model (**c, d**) for predicting MI events and non-events with or without the clinical model. Panels **a** and **c** show reclassification with the specific protein model alone, while panels **b** and **d** show the reclassification when the clinical model is included. Reclassification was assessed using a 9.0% predicted 10-year MI risk observed in this subpopulation for the net reclassification index (NRI) analysis. CI, confidence interval

To further assess real-world reclassification performance, we evaluated the reclassification performance in a high-risk population (male smokers with elevated SBP and non-HDL cholesterol), integrating protein markers identified from subgroup-specific models. The protein panel significantly improved categorical risk stratification (NRI = + 0.125 [0.026–0.228], *P* = 0.015) by correctly reclassifying individuals with incident MI (event NRI = + 0.309 [0.214–0.407], *P* < 0.001) (Fig. 5a). Importantly, the inclusion of the clinical model did not result in further improvement (Fig. 5b), indicating that the protein panel itself was the key contributor to enhanced classification. The specific protein model also outperformed the general protein model, particularly in the accurate classification of non-MI individuals (non-event NRI = + 0.030 [0.008–0.054], *P* = 0.010) (Fig. 5c, d). A comparable analysis incorporating individuals with diabetes into the high-risk definition yielded similar improvements in reclassification (Fig. S8). To further evaluate the reliability of this modeling approach, we assessed reclassification performance across all 32 SCORE2-derived clinical subgroups (Supplementary Table S4). This analysis revealed that 7 out of 32 subgroups showed statistically significant gains in overall reclassification, particularly among individuals with multiple risk factors (Supplementary Table S14). Moreover, 18 of the 32 subgroups demonstrated improved discrimination of individuals who remained MI-free (Supplementary Table S14). A similar pattern was also observed when comparing the subgroup-specific and general protein models (Supplementary Table S15).

## Discussion

In this large-scale proteomic analysis using UK Biobank, we demonstrated that integrating plasma protein profiles with SCORE2 stratification variables substantially improved the prediction of incident MI. By developing a subgroup-specific prediction model, we identified distinct and clinically meaningful proteomic signatures that significantly outperformed models based solely on SCORE2-aligned clinical predictors in each subgroup. Notably, the predictive performance of the subgroup-specific protein panel was greater than that of the clinical panel in high-risk individuals.

Given the well-established heterogeneity in the pathophysiology of CVDs [31], we hypothesized that proteomic predictors of MI would vary across clinically defined subgroups. This hypothesis was supported by our findings, with over half of the predictive proteins (17 out of 29) being uniquely identified in one of the specific subgroups. Building on the established SCORE2 stratification framework, we stratified over 50,000 participants into 10 clinical subgroups and developed the SCORE2-Pro model. This approach represents a step forward toward personalized risk prediction using large-scale proteomic data, consistent with the previous findings showing that sex-specific proteomic panels improved SCORE2-based prediction of MACE [13]. Notably, our SCORE2-Pro is a de novo machine-learning model trained using SCORE2 covariates together with plasma proteomics, rather than incorporating the published SCORE2 equation or coefficients. Although our analysis included a large proteomic cohort (n = 51,010), the sample size was still insufficient to reproduce the full granularity of SCORE2 risk stratification, particularly the multiple discrete bands for SBP and non-HDL cholesterol. Therefore, we adopted a binary-split categorization strategy for these variables. The reliability of this approach was subsequently assessed through a systematic evaluation across all 32 stratified subgroups using calibrated performance metrics (calibrated NRI, Supplementary Table S14–S15).

Recent studies showed that inclusion of large proteomic panels into clinical risk models yields only modest performance gains in the general population. A recent study reported that a 70-protein score improved AUC by only 0.014 for predicting incident CVDs [11]. Similarly, an analysis by Royer et al. using the full UK Biobank proteomic dataset showed an AUC improvement of 0.031 for predicting MACE [10]. We observed a similar AUC gain of 0.033 for MI in the overall population. However, when focusing on stratified subgroups, the improvement was consistently greater, with the highest gain observed in individuals with non-HDL cholesterol < 4 mmol/L (AUC + 0.094). This suggests that subgroup-specific proteomic modeling may improve the detection of real biological signals in each subgroup. We also observed stronger discriminative performance of proteomic models in higher-risk subgroups (Supplementary Table S14–S15). This is consistent with recent evidence suggesting that the added predictive value of proteomic markers is more pronounced in individuals at higher risk [10, 11]. The greater effectiveness of protein-based models in secondary prevention than primary prevention further supports this concept [11, 12, 32–34].

GDF15, a cytokine responsive to inflammatory and metabolic stress [35, 36], has been widely reported as a key predictor of CVDs [7, 12, 13]. Deng et al. further identified GDF15 as one of the most pleiotropic disease-associated proteins [37]. In line with prior evidence, GDF15 was identified as a key predictor in 9 of 10 subgroups, highlighting its robust predictive value across diverse clinical subgroups. Interestingly, GDF15 was not selected in the middle-aged subgroup and did not even rank among the top 20 proteins by importance. This may be explained by age-dependent myocardial inflammation, which influences the progression of heart diseases [38]. MMP12 was similarly identified as a consistently important predictor across all subgroups, likely due to its central role in vascular remodeling and inflammation across diverse clinical profiles. In contrast, Renin (REN) was uniquely identified in the middle-aged subgroup, possibly due to age-related changes in renal function and renin-angiotensin system activity [40]. In addition, ADGRG2 emerged as the only protective marker in the prediction model among the general population and was found in the female subgroup. This gene is located on the X chromosome and has been linked to male fertility, suggesting a potential role of ADGRG2 in sex-related differences in MI risk [41, 42]. Overall, the subgroup-specific protein panels revealed in this study may reflect distinct biological pathways underlying MI in different populations, offering mechanistic insights for future investigation.

Our study has provided a clear example of how proteomic data can enhance clinical prediction and personalized care. We identified subgroup-specific proteins that improve the accuracy of MI risk prediction, offering a more tailored approach to prevention. For example, individuals traditionally classified as low-risk by SCORE2, such as younger adults or those with lower non-HDL cholesterol, exhibited distinct proteomic profiles for MI prediction. Conversely, in high-risk populations such as male smokers with elevated SBP, the proteomic integration significantly improved event prediction and risk stratification.

This study has several strengths. First, we leveraged the large-scale and deeply phenotyped UK Biobank, enabling robust stratified analyses across clinically relevant subgroups with adequate statistical power. Second, our SCORE2-Pro model, a subgroup-stratified plasma proteomic prediction framework, was designed to mirror the clinical stratification structure of SCORE2. Third, the predictive models were developed using a well-validated nonlinear machine learning approach (light gradient boosting) [26], and the protein panels were selected through a forward selection strategy based on ranked importance. We deliberately selected the minimal set of proteins that maximized predictive performance, thereby enhancing both the interpretability and translational feasibility of the resulting models.

Nonetheless, several limitations need to be considered. First, our findings are based on internal hold-out validation within the UK Biobank. However, given the large sample size of UKB-PPP, this validation strategy was applied in several other studies using the same data [9, 10]. Second, the proteomic data were generated using the Olink panel [23], which limits the range of proteins; broader platforms may reveal additional informative biomarkers. Third, the UK Biobank participants are predominantly of white British and generally considered low-risk under SCORE2; although individuals of other ancestries were included, we were underpowered to stratify the model by ethnicity or region. Fourth, individuals with diabetes were included in our analyses, whereas SCORE2 excludes diabetic participants and instead uses the separate SCORE2-Diabetes algorithm for risk estimation. Although sensitivity analyses showed that the proteomic model remained robust when diabetic individuals were incorporated into the high-risk group, the relatively small number of participants with diabetes limited our ability to develop a diabetes-specific proteomic model analogous to SCORE2-Diabetes. Finally, baseline use of cardiovascular medications (e.g., statins) was not fully adjusted for, which might influence both protein levels and event risk.

In conclusion, we identified clinically stratified, proteomic signatures that enhance MI risk prediction beyond the traditional clinical models. By aligning with SCORE2-defined subgroups and applying robust machine learning, we developed the SCORE2-Pro model, which revealed biologically distinct and practically relevant protein panels in ten subgroups. These results illustrate the potential of precision proteomics to refine cardiovascular risk assessment and guide individualized preventive strategies.

## Supplementary Information

Supplementary file1.

Supplementary file2.

## Data Availability

Because of the sensitive nature of the data, access is restricted to qualified researchers who have received training in human subject confidentiality. Data requests can be submitted to UK Biobank via [https://www.ukbiobank.ac.uk] (https:/www.ukbiobank.ac.uk).
