# Supplementary material for: Clinical subgroup-stratified plasma proteomic signatures improve risk prediction for myocardial infarction: SCORE2-Pro
Source: Cardiovasc Diabetol. 2026 Feb 2;25:70. doi: 10.1186/s12933-025-03050-7 (PMC12951900; doi:10.1186/s12933-025-03050-7)
Supplement: Supplementary file 1 — Supplementary file1. [file 12933_2025_3050_MOESM1_ESM.pdf]

Figure S1

a

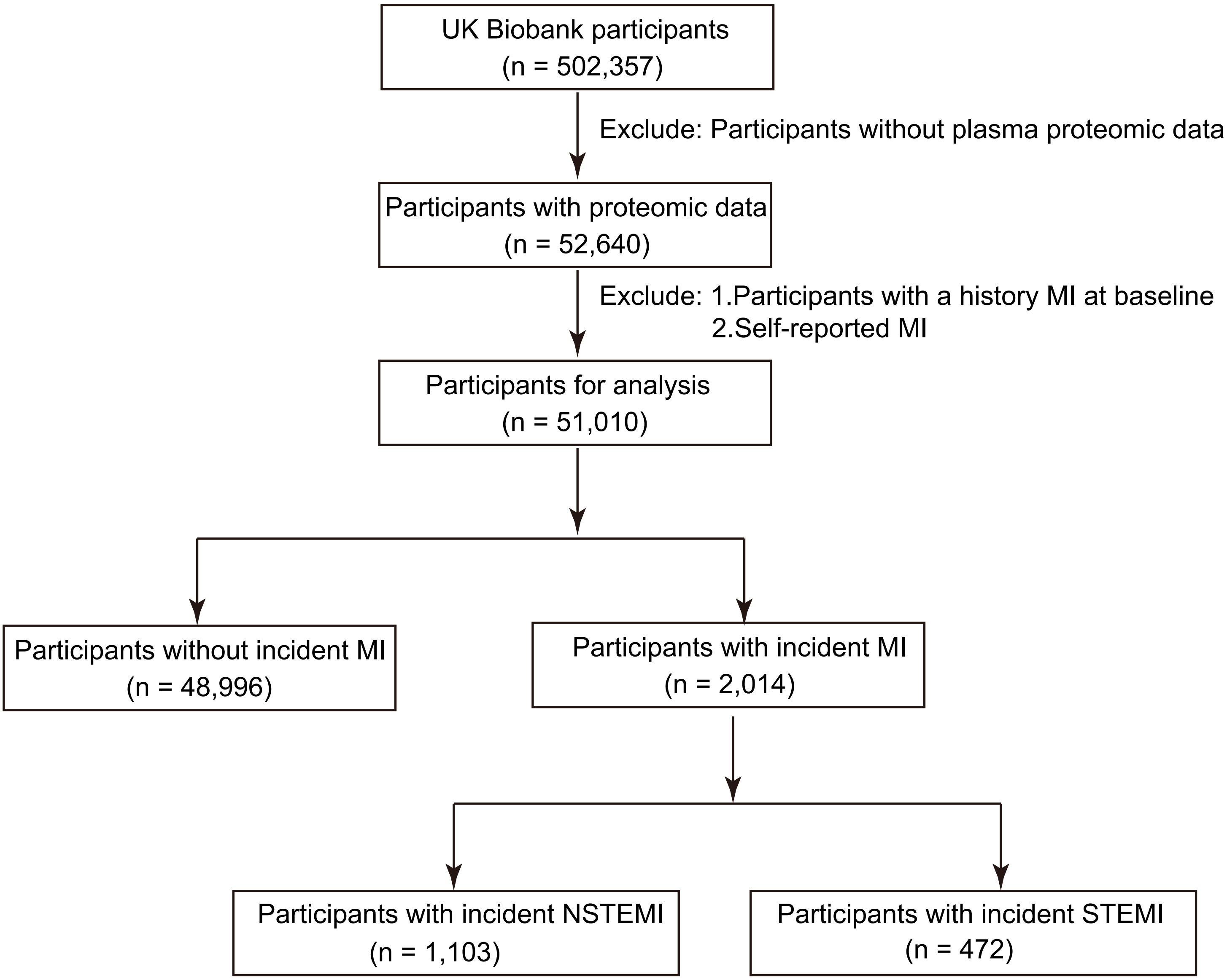
UK Biobank participants
(n = 502,357)
Exclude: Participants without plasma proteomic data
Participants with proteomic data
(n = 52,640)
Exclude: 1.Participants with a history MI at baseline
2.Self-reported MI
Participants for analysis
(n = 51,010)
Participants without incident MI
(n = 48,996)
Participants with incident MI
(n = 2,014)
Participants with incident NSTEMI
(n = 1,103)
Participants with incident STEMI
(n = 472)

Figure S1. Flowchart showing the enrollment of the study participants.

We analyzed 51,010 participants with proteomic data. Participants had no diagnosis or reports of MI at baseline. MI, myocardial infarction; NSTEMI, non-ST-elevation MI; STEMI, ST-elevation MI

Figure S2

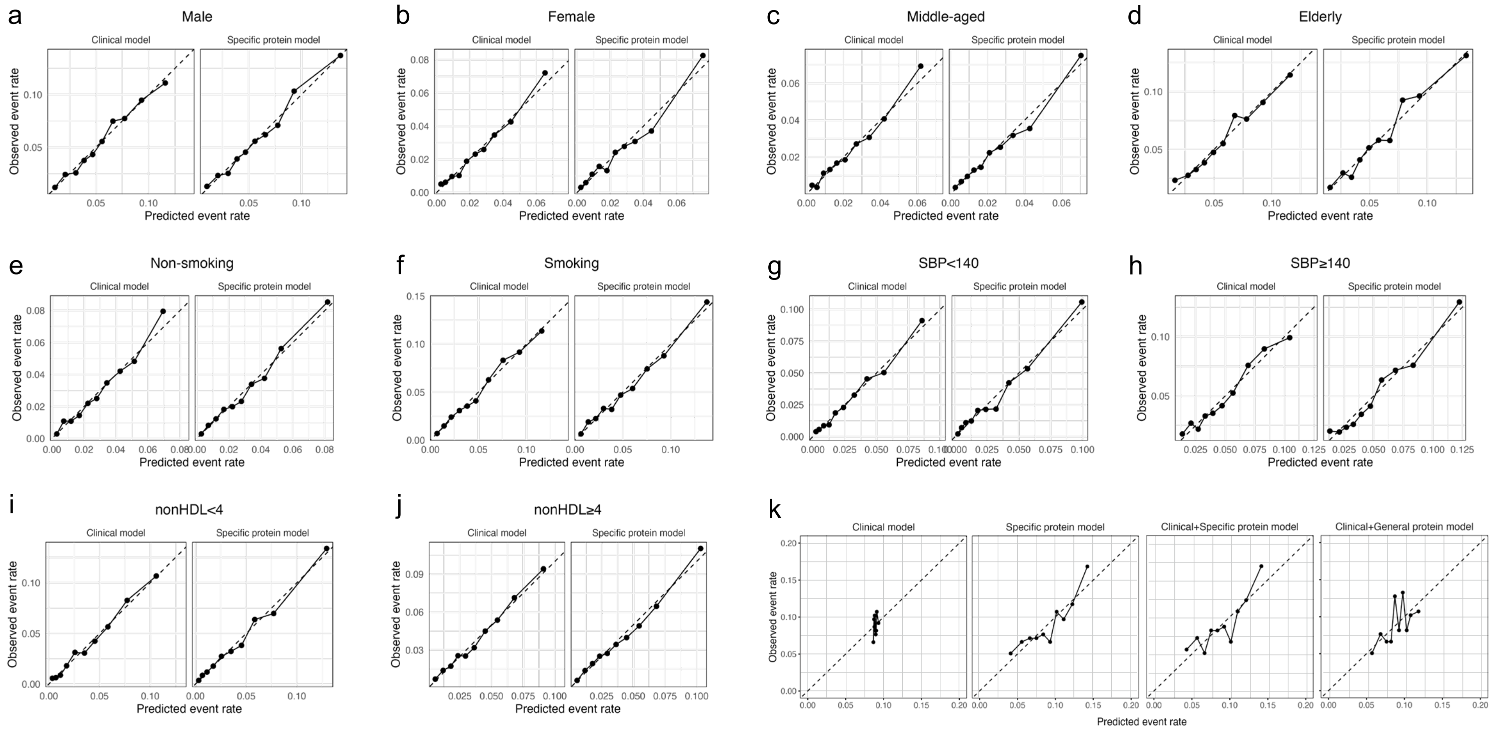
a
Male
b
Female
c
Middle-aged
d
Elderly
e
Non-smoking
f
Smoking
g
SBP<140
h
SBP≥140
i
nonHDL<4
j
nonHDL≥4
k
Clinical model
Specific protein model
Clinical+Specific protein model
Clinical+General protein model
Observed event rate
Predicted event rate

Figure S2. Calibration performance of clinical and subgroup-specific protein models.

Calibration curves of the clinical and subgroup-specific protein models for MI risk prediction across stratified clinical subgroups (a–j) and high-risk population (male, elderly smokers with elevated SBP and non-HDL cholesterol) (k).

Figure S3

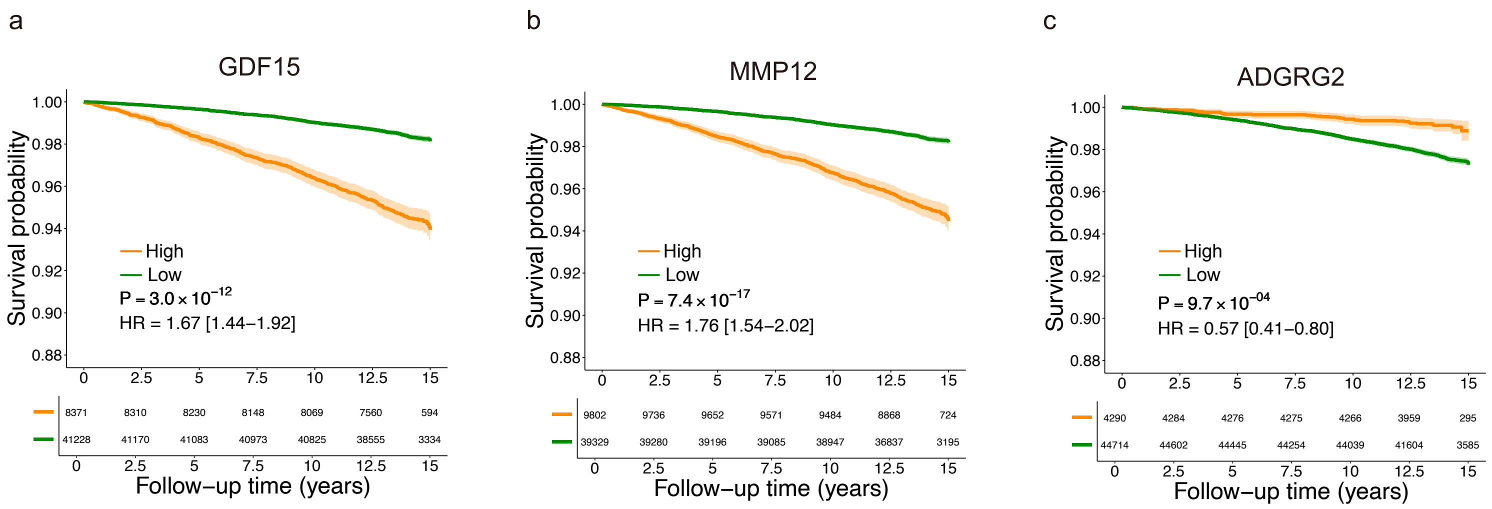
a
GDF15
Survival probability
High
Low
P = 3.0 × 10^−12
HR = 1.67 [1.44–1.92]
8371 8310 8230 8148 8069 7560 594
41228 41170 41083 40973 40825 38555 3334
Follow−up time (years)
b
MMP12
Survival probability
High
Low
P = 7.4 × 10^−17
HR = 1.76 [1.54–2.02]
9802 9736 9652 9571 9484 8868 724
39329 39280 39196 39085 38947 36837 3195
Follow−up time (years)
c
ADGRG2
Survival probability
High
Low
P = 9.7 × 10^−04
HR = 0.57 [0.41–0.80]
4290 4284 4276 4275 4266 3959 295
44714 44602 44445 44254 44039 41604 3585
Follow−up time (years)

Figure S3. Kaplan-Meier curves for NSTEMI incidence.

Unadjusted Kaplan-Meier curves show NSTEMI incidence over time. The numbers presented below each curve represent the number of participants remaining at risk at the corresponding follow-up time points. Cox models adjusted for age, sex, and education were used to estimate the association between baseline protein levels and MI risk. Shaded regions represent standard errors.

Figure S4

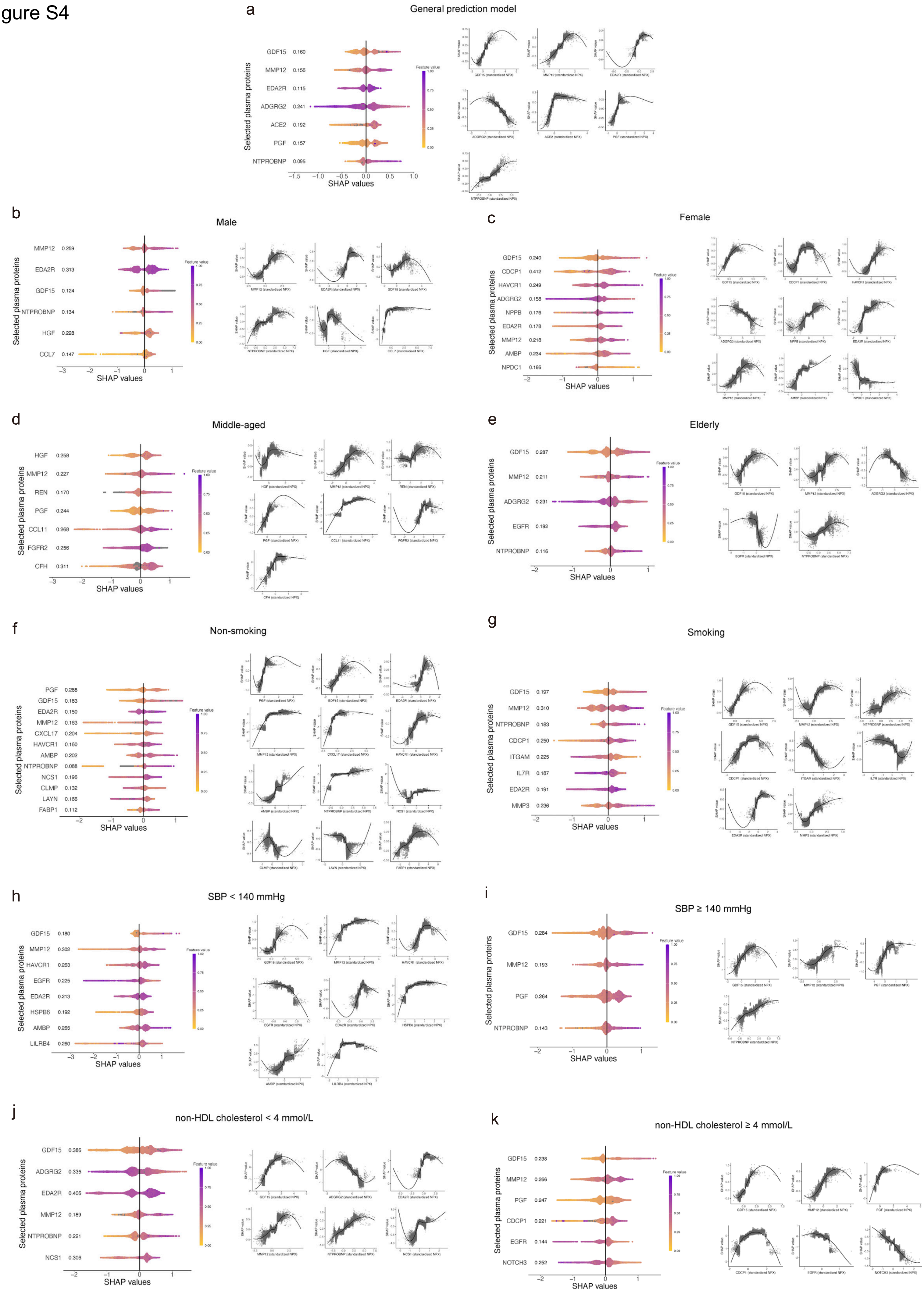
a
General prediction model
Selected plasma proteins
GDF15 0.160
MMP12 0.156
EDA2R 0.115
ADGRG2 0.241
ACE2 0.192
PGF 0.157
NTPROBNP 0.095
SHAP values
Feature value
b
Male
MMP12 0.259
EDA2R 0.313
GDF15 0.124
NTPROBNP 0.134
HGF 0.228
CCL7 0.147
c
Female
GDF15 0.240
CDCP1 0.412
HAVCR1 0.249
ADGRG2 0.158
NPPB 0.176
EDA2R 0.178
MMP12 0.218
AMBP 0.234
NPDC1 0.166
d
Middle-aged
HGF 0.258
MMP12 0.227
REN 0.170
PGF 0.244
CCL11 0.268
FGFR2 0.256
CFH 0.311
e
Elderly
GDF15 0.287
MMP12 0.211
ADGRG2 0.231
EGFR 0.192
NTPROBNP 0.116
f
Non-smoking
PGF 0.288
GDF15 0.183
EDA2R 0.150
MMP12 0.163
CXCL17 0.204
HAVCR1 0.160
AMBP 0.202
NTPROBNP 0.088
NCS1 0.196
CLMP 0.132
LAYN 0.166
FABP1 0.112
g
Smoking
GDF15 0.197
MMP12 0.310
NTPROBNP 0.183
CDCP1 0.250
ITGAM 0.225
IL7R 0.187
EDA2R 0.191
MMP3 0.236
h
SBP < 140 mmHg
GDF15 0.180
MMP12 0.302
HAVCR1 0.263
EGFR 0.225
EDA2R 0.213
HSPB6 0.192
AMBP 0.265
LILRB4 0.260
i
SBP ≥ 140 mmHg
GDF15 0.284
MMP12 0.193
PGF 0.264
NTPROBNP 0.143
j
non-HDL cholesterol < 4 mmol/L
GDF15 0.386
ADGRG2 0.335
EDA2R 0.406
MMP12 0.189
NTPROBNP 0.221
NCS1 0.306
k
non-HDL cholesterol ≥ 4 mmol/L
GDF15 0.238
MMP12 0.266
PGF 0.247
CDCP1 0.221
EGFR 0.144
NOTCH3 0.252

Figure S4. SHAP visualization of general and subgroup-specific protein models in MI prediction.

The SHapley Additive exPlanations (SHAP) plots (left) show the relative contribution of each protein to MI risk prediction, with wider distributions indicating stronger influence on the model. Colors represent protein expression levels, from low (yellow) to high (purple). The dependence plots (right) illustrate the functional relationship between protein levels and their SHAP values, highlighting how changes in protein concentrations affect predicted MI risk.

Figure S5

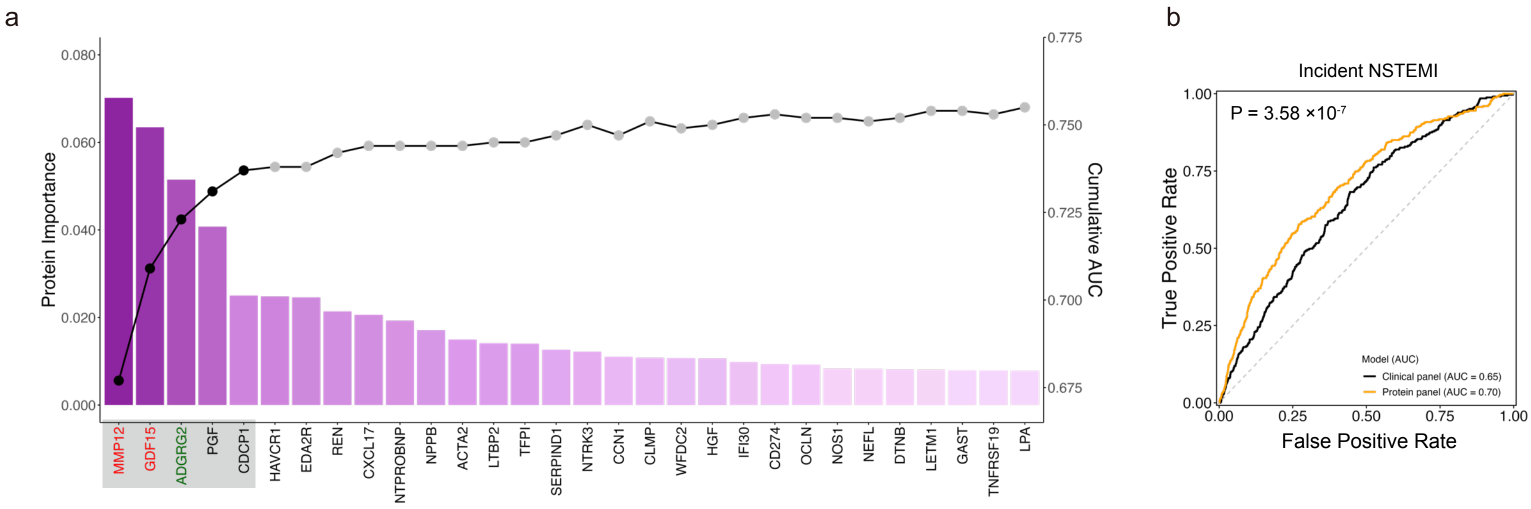
a
Protein Importance
Cumulative AUC
0.080
0.060
0.040
0.020
0.000
0.775
0.750
0.725
0.700
0.675
MMP12
GDF15
ADGRG2
PGF
CDCP1
HAVCR1
EDA2R
REN
CXCL17
NTPROBNP
NPPB
ACTA2
LTBP2
TFPI
SERPIND1
NTRK3
CCN1
CLMP
WFDC2
HGF
IFI30
CD274
OCLN
NOS1
NEFL
DTNB
LETM1
GAST
TNFRSF19
LPA
b
Incident NSTEMI
P = 3.58 ×10-7
True Positive Rate
False Positive Rate
1.00
0.75
0.50
0.25
0.00
0.00
0.25
0.50
0.75
1.00
Model (AUC)
Clinical panel (AUC = 0.65)
Protein panel (AUC = 0.70)

Figure S5. Importance ranking of proteins in the NSTEMI prediction model.

(a) Sequential forward selection from candidate proteins in the NSTEMI model in the derivation set. The bar plot illustrates the importance of sorted proteins based on their contributions to predicting future MI (left axis). The line plot shows cumulative Area Under the Curve (AUC) values (right axis) as proteins are included one by one in each iteration. Five proteins selected for the subsequent NSTEMI prediction model are highlighted with gray rectangular backgrounds.

(b) Receiver operating characteristic (ROC) curves and AUC values show the performance of different variable models for predicting incident NSTEMI in the validation set. The protein panel includes top-ranked five proteins identified in Fig. S3a.

Figure S6

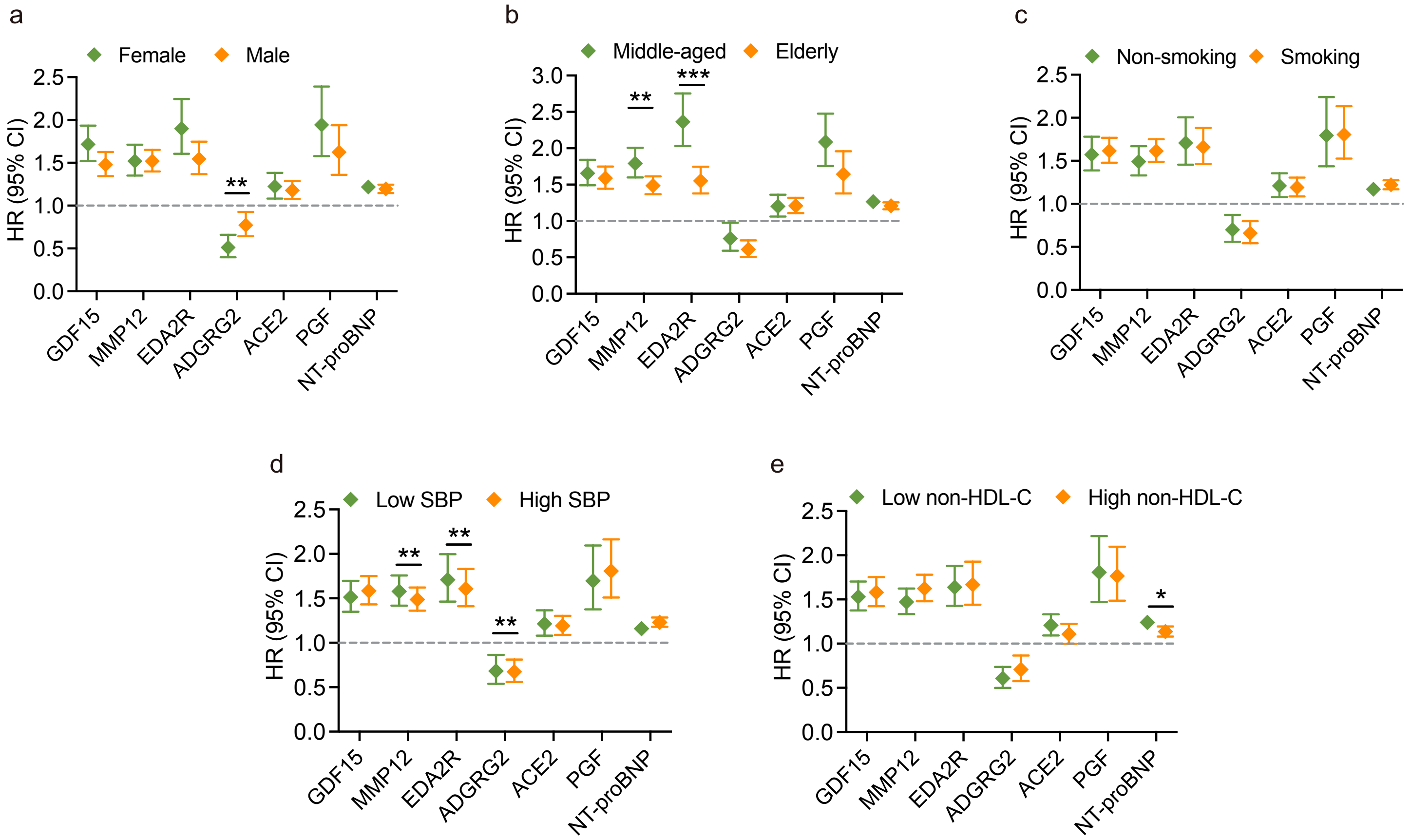

a
Female
Male
HR (95% CI)
2.5
2.0
1.5
1.0
0.5
0.0
**
GDF15
MMP12
EDA2R
ADGRG2
ACE2
PGF
NT-proBNP
b
Middle-aged
Elderly
3.0
**
***
c
Non-smoking
Smoking
d
Low SBP
High SBP
e
Low non-HDL-C
High non-HDL-C
*

Figure S6. Subgroup-specific association between seven top-performing proteins and myocardial infarction.

Hazard ratios (HR) with 95% confidence intervals (CI) of the seven top-ranked proteins for myocardial infarction in subgroup populations. The models were adjusted for diabetes diagnosis, smoking status, body mass index, SBP, diastolic blood pressure, total cholesterol, triglycerides, HDL cholesterol, LDL cholesterol, C-reactive protein, and estimated glomerular filtration rate. Pairwise Z-tests were used to compare HR values between groups. Statistical significance is denoted as follows: * $P < 0.05$, ** $P < 0.01$, *** $P < 0.001$.

Figure S7

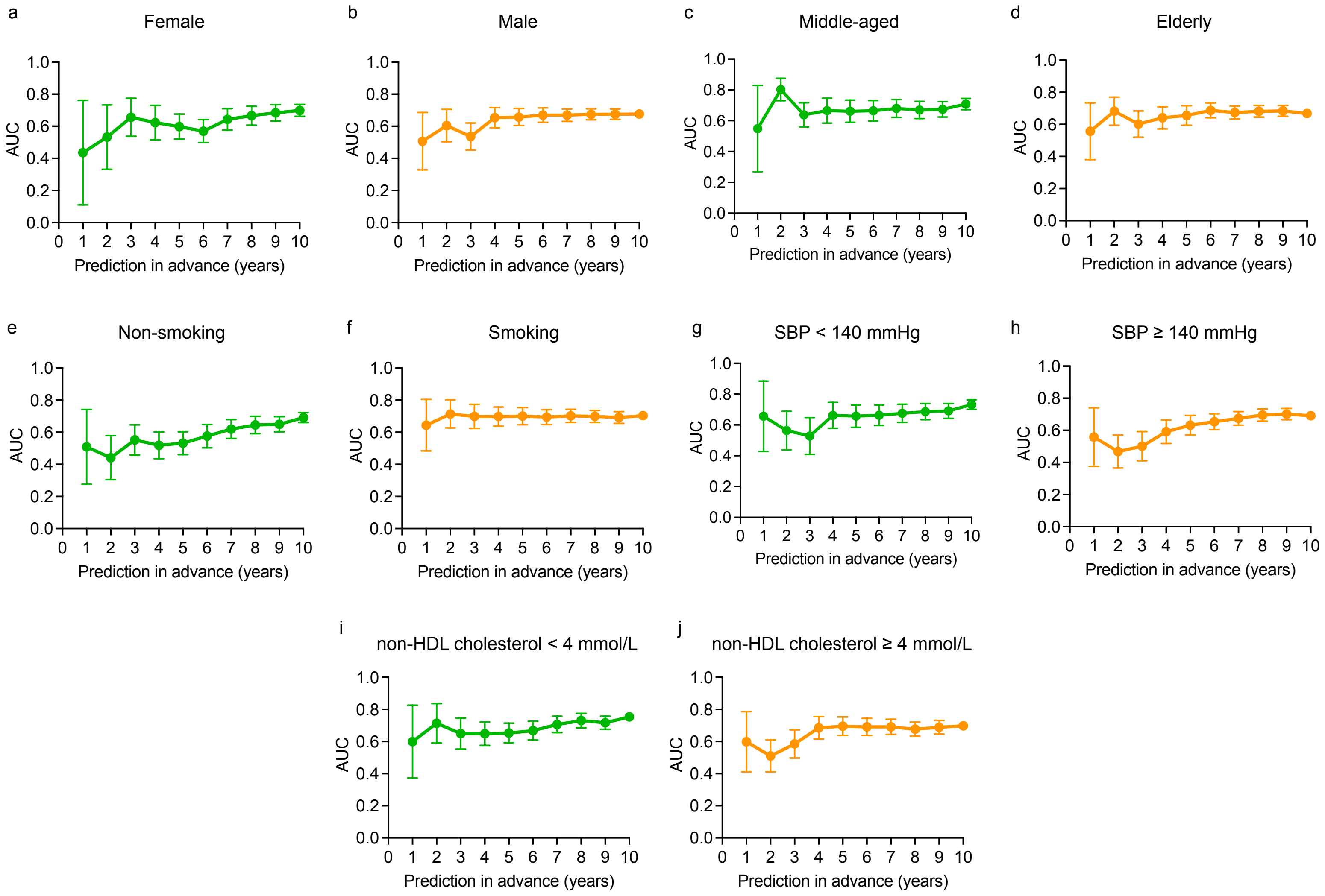
a
Female
b
Male
c
Middle-aged
d
Elderly
e
Non-smoking
f
Smoking
g
SBP < 140 mmHg
h
SBP ≥ 140 mmHg
i
non-HDL cholesterol < 4 mmol/L
j
non-HDL cholesterol ≥ 4 mmol/L
AUC
0.0
0.2
0.4
0.6
0.8
1.0
0 1 2 3 4 5 6 7 8 9 10
Prediction in advance (years)

Figure S7. Dynamic AUC trajectories across clinical subgroups.

Line plots of the subgroup-specific dynamic AUC for predicting incident MI over time in the validation set.

Figure S8

Correctly reclassified (green) | Incorrectly reclassified (orange)

a

	Clinical model	Specific protein model: Low risk	Specific protein model: High risk	Total No. of participants (%)
MI events	Low risk	73	93	166 (37.7)
MI events	High risk	78	196	274 (62.3)
MI events	Total No. of participants (%)	151 (34.3)	289 (65.7)	440 (100)
Free of MI events	Low risk	1286	655	1941 (46.7)
Free of MI events	High risk	1121	1097	2218 (53.3)
Free of MI events	Total No. of participants (%)	2407 (57.9)	1752 (42.1)	4159 (100)

NRI [95% CI] P value
Total: 0.146 [0.085 – 0.296] P = 0.007
MI events: 0.034 [-0.027 – 0.093] P = 0.27
MI noevents: 0.112 [0.091 – 0.130] P < 0.001

b

	Clinical model	Clinical model + Specific protein model: Low risk	Clinical model + Specific protein model: High risk	Total No. of participants (%)
MI events	Low risk	77	89	166 (37.7)
MI events	High risk	76	198	274 (62.3)
MI events	Total No. of participants (%)	153 (34.8)	287 (65.2)	440 (100)
Free of MI events	Low risk	1363	578	1941 (46.7)
Free of MI events	High risk	1084	1134	2218 (53.3)
Free of MI events	Total No. of participants (%)	2447 (58.8)	1712 (41.2)	4159 (100)

NRI [95% CI], P value
Total: 0.151 [0.091 – 0.213], P < 0.001
MI events: 0.030 [-0.030 – 0.088], P = 0.32
MI noevents: 0.122 [0.102 – 0.140], P < 0.001

c

	General protein model	Specific protein model: Low risk	Specific protein model: High risk	Total No. of participants (%)
MI events	Low risk	116	49	165 (37.7)
MI events	High risk	35	240	275 (62.5)
MI events	Total No. of participants (%)	151 (34.3)	289 (65.7)	440 (100)
Free of MI events	Low risk	1948	427	2375 (57.1)
Free of MI events	High risk	459	1325	1784 (42.9)
Free of MI events	Total No. of participants (%)	2407 (57.9)	1752 (42.1)	4159 (100)

NRI [95% CI], P value
Total: 0.040 [-0.003 – 0.087], P = 0.08
MI events: 0.032 [-0.009 – 0.076], P = 0.14
MI noevents: 0.008 [-0.007 – 0.022], P = 0.28

d

	Clinical model + General protein model	Clinical model + Specific protein model: Low risk	Clinical model + Specific protein model: High risk	Total No. of participants (%)
MI events	Low risk	123	40	163 (37.0)
MI events	High risk	30	247	277 (63.0)
MI events	Total No. of participants (%)	153 (34.8)	287 (65.2)	440 (100)
Free of MI events	Low risk	1991	357	2348 (56.5)
Free of MI events	High risk	456	1355	1811 (43.5)
Free of MI events	Total No. of participants (%)	2447 (58.8)	1712 (41.2)	4159 (100)

NRI [95% CI], P value
Total: 0.047 [0.008 – 0.087], P = 0.02
MI events: 0.023 [-0.014 – 0.061], P = 0.23
MI noevents: 0.024 [0.010 – 0.037], P < 0.001

Figure S8. Reclassification in the high-risk population including individuals with diabetes.

The figure shows reclassification results in the extended high-risk population (male, elderly smokers with elevated SBP and non-HDL cholesterol, and/or diagnosed diabetes). The reclassification tables compare the performance of SCORE2-Pro (a-b) and the general protein model (c-d) for predicting MI events and non-events with or without the clinical model. Panels (a) and (c) show reclassification with the specific protein model alone, while panels (b) and (d) show the reclassification when the clinical model is included. Reclassification was assessed using a 9.5% predicted 10-year MI risk observed in this subpopulation for the NRI analysis. CI, confidence interval.
